# Bilateral Symptomatic Cystic Lesions of the Palatine Tonsils Successfully Treated by Endoscopic Excision: A Rare Case Report

**DOI:** 10.1155/crot/4153080

**Published:** 2026-04-12

**Authors:** Tatsuya Masaki, Junko Tsuda, Youhei Yamamoto, Shogo Nishimura, Yosuke Okinaka, Mei Sakamoto, Yosuke Takemoto, Toshiki Masumitsu, Makoto Hashimoto, Kazuma Sugahara

**Affiliations:** ^1^ Department of Otolaryngology, Yamaguchi University Graduate School of Medicine, Ube, 755-8505, Yamaguchi, Japan, yamaguchi-u.ac.jp

**Keywords:** bilateral, endoscopic excision, palatine tonsil, tonsillar cyst

## Abstract

Cystic lesions of the palatine tonsils may occasionally be encountered in clinical practice, whereas bilateral involvement is exceptionally uncommon. We report the case of a 74‐year‐old man who presented with pharyngeal discomfort and bilateral cystic lesions in the inferior poles of the palatine tonsils. Endoscopic examination and radiological imaging revealed well‐circumscribed cystic lesions without diffusion restriction. The lesions were excised endoscopically under general anesthesia, and histopathological examination revealed cyst walls lined by stratified squamous epithelium with underlying lymphoid tissue. The postoperative course was uneventful, and no recurrence was observed during the 1‐year follow‐up period.

## 1. Introduction

Cystic lesions of the palatine tonsils may occasionally be encountered in clinical practice, including epidermoid, lymphoepithelial, and retention cysts [1–3]. Most reported cases are unilateral, and bilateral involvement of the palatine tonsils is exceptionally uncommon [4].

Clinically, these lesions may be asymptomatic or present with nonspecific symptoms, such as pharyngeal discomfort. Endoscopic findings often show smooth‐surfaced, well‐circumscribed lesions that can mimic tonsillar tumors, making preoperative diagnosis challenging [5–7].

Radiological imaging is useful for evaluating cystic morphology; however, a definitive diagnosis requires histopathological examination [8–10]. Here, we report a rare case of bilateral palatine tonsillar cysts with detailed clinical, radiological, surgical, and pathological findings.

## 2. Case Presentation

A 74‐year‐old man presented with a 1‐month history of pharyngeal discomfort during swallowing.

He had no history of smoking or alcohol consumption. His medical history included surgical treatment of childhood empyema, appendectomy for peritonitis, arthritis requiring hospitalization, and paroxysmal atrial fibrillation with bradycardia. He had documented allergies to sulfonamides and contrast agents. He had no history of recurrent tonsillitis or pharyngitis.

### 2.1. Examinations

**FIGURE 1:**
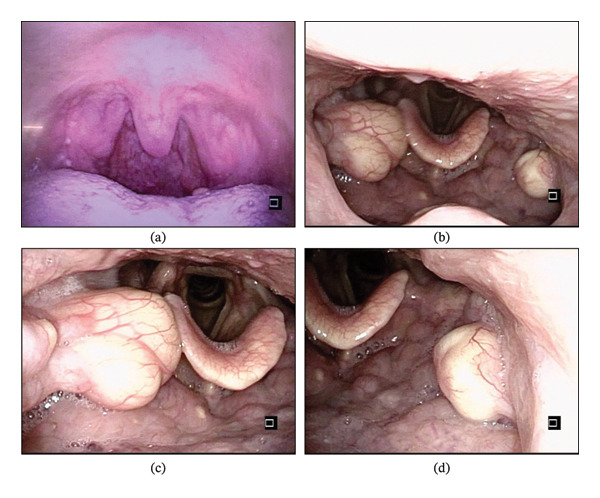
Oropharyngeal and endoscopic findings of the palatine tonsillar cysts. (a) Oropharyngeal examination reveals a subtle cystic lesion in the inferior pole of the left palatine tonsil. (b) Endoscopic view at the oropharyngeal level demonstrates smooth‐surfaced, cream‐colored, spherical masses located in the inferior poles of both palatine tonsils. (c) Magnified endoscopic view of the right palatine tonsil shows a well‐circumscribed cystic lesion with a smooth surface. (d) Magnified endoscopic view of the left palatine tonsil demonstrates a similar cystic lesion.

Oropharyngeal examination revealed a subtle cystic lesion in the inferior pole of the left palatine tonsil. Endoscopic evaluation revealed smooth‐surfaced, cream‐colored, spherical masses at the inferior poles of both palatine tonsils (Figure 1).

**FIGURE 2:**
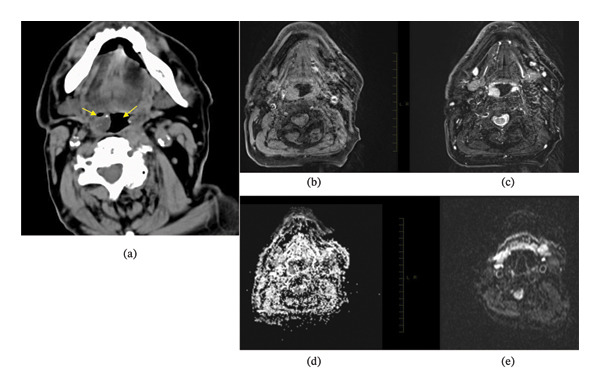
Radiological findings of the palatine tonsillar cysts. (a) Axial computed tomography at the oropharyngeal level demonstrates bilateral, spherical soft‐tissue masses protruding into the oropharyngeal lumen. (b) Axial T1‐weighted magnetic resonance image shows the lesions with an isointense signal to the surrounding soft tissue. (c) Axial T2‐weighted magnetic resonance image reveals high signal intensity of the lesions, consistent with cystic contents. (d) Apparent diffusion coefficient (ADC) map shows no high signal intensity. (e) Diffusion‐weighted imaging demonstrates the absence of restricted diffusion. The masses identified on computed tomography appeared isointense on T1‐weighted images and hyperintense on T2‐weighted images, without hyperintense signals on diffusion‐weighted images or the ADC map.

Noncontrast axial computed tomography showed bilateral spherical soft tissue masses protruding into the oropharyngeal lumen at the oropharyngeal level (Figure 2(a)). Magnetic resonance imaging was performed without contrast because of allergy to the contrast agent. The lesions were isointense on T1‐weighted images and hyperintense on T2‐weighted images (Figures 2(b) and 2(c)). Diffusion‐weighted imaging and the apparent diffusion coefficient map showed the absence of restricted diffusion (Figures 2(d) and 2(e)). These findings suggested cystic lesions; however, neoplastic processes could not be completely excluded.

### 2.2. Treatment and Clinical Course

**FIGURE 3:**
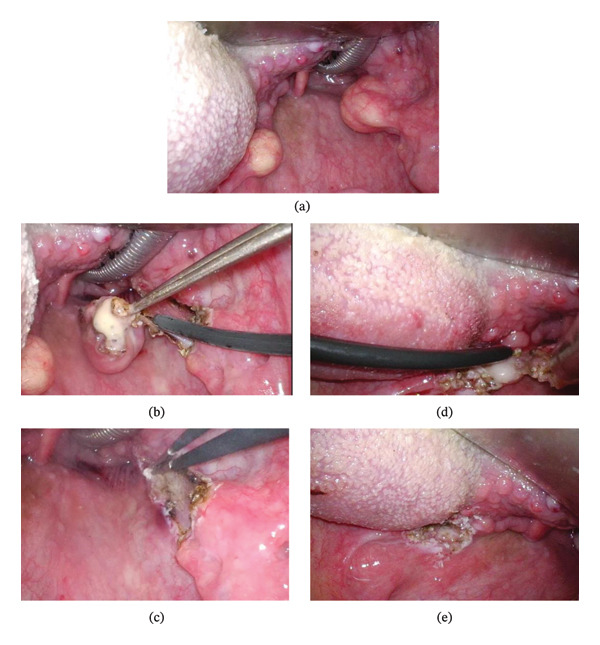
Intraoperative findings during endoscopic excision. (a) Intraoperative view after laryngeal exposure. (b) Endoscopic excision of the cyst of the right palatine tonsil using a radiofrequency knife. (c) Surgical field after complete excision of the right‐sided cyst. (d) Endoscopic excision of the cyst of the left palatine tonsil using a radiofrequency knife. (e) Surgical field after complete excision of the left‐sided cyst.

The patient did not report clear lateralization of the pharyngeal discomfort. Although the lesion on the left side appeared relatively small, bilateral excision was planned to obtain a definitive diagnosis and to prevent possible future enlargement. Given the patient’s symptoms, the possibility of progressive enlargement, and the strong preference for surgical treatment, endoscopic excision was planned under general anesthesia. After laryngeal exposure using a retractor, the cysts were endoscopically excised using a radiofrequency knife. The right cyst was removed first, followed by the left cyst. Hemostasis was achieved using bipolar coagulation (Figure 3).

**FIGURE 4:**
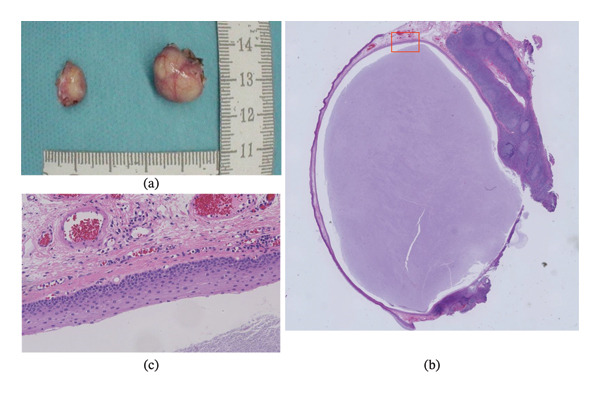
Gross and histopathological findings of the excised specimens. (a) Gross appearance of the excised cystic specimen. (b) Low‐power view of the histopathological section stained with hematoxylin and eosin, showing the overall architecture of the cystic lesion. (c) High‐power view of the boxed area in (b). The cyst wall is lined with stratified squamous epithelium, and intraluminal deposits are observed. Subepithelial lymphoid tissue with lymphocytic aggregation, consistent with tonsillar tissue, is identified.

Gross examination revealed well‐circumscribed cystic lesions. Histopathologically, the cyst walls were lined with stratified squamous epithelium with intraluminal deposits. Subepithelial lymphoid tissue with lymphocytic aggregation consistent with tonsillar tissue was identified; however, no malignant features were observed (Figure 4). Based on these findings, the lesions were diagnosed as bilateral retention cysts of the palatine tonsils. No histopathological differences were observed between the right and left lesions.

The postoperative course was uneventful until Postoperative Day 6, when minor bleeding occurred from the left inferior tonsillar pole. Bleeding resolved with conservative management, and the patient was discharged without further complications. At the 1‐year follow‐up, no recurrence was observed, and the patient reported resolution of the pharyngeal discomfort.

## 3. Discussion

Cystic lesions of the palatine tonsils may occasionally be encountered in clinical practice, and most reported cases involve unilateral disease. However, bilateral involvement of the palatine tonsils is extremely uncommon. Epidermoid, lymphoepithelial, and retention cysts are the main pathological entities described in the literature; however, clinically apparent tonsillar cystic lesions are rare [1–3, 11, 12].

Histopathological examinations of routinely excised palatine tonsils have revealed that microscopic cystic changes are frequently observed in tonsillectomy specimens, particularly in patients with chronic inflammation. Cysts are typically small incidental findings and are clinically insignificant. In contrast, cystic lesions that grow large enough to become clinically apparent or cause symptoms are relatively uncommon. The present case falls into this category and is, therefore, of clinical interest.

Bilateral involvement of the palatine tonsils is rare. To date, only a few cases have been reported in the international literature, underscoring the exceptional rarity of this case [4]. Reporting such rare cases is important to better characterize their clinical features and optimize management strategies.

To further contextualize the rarity of the present case, previously reported palatine tonsillar cysts are summarized in Table 1 [1–6, 8–16]. Most of the reported cases involved unilateral lesions, with epidermoid cysts being the most common and lymphoepithelial cysts and retention cysts as less frequent types. Bilateral involvement of the palatine tonsils has only rarely been documented, with only a few reports describing bilateral lymphoepithelial cysts [4, 14].

**TABLE 1:** International reports of palatine tonsil cysts.

No.	Author	Year	Country	Cyst type	Laterality
1	Tanaka et al.	2004	Japan	Lymphoepithelial cyst	Unilateral
2	Erol et al.	2013	Turkey	Epidermoid cyst	Unilateral
3	Mohsenifar et al.	2013	Iran	Lymphoepithelial cyst	Bilateral
4	Castro et al.	2015	Brazil	Lymphoepithelial cyst	Unilateral
5	Gulia et al.	2015	India	Epidermoid cyst	Unilateral
6	Bingöl et al.	2016	Turkey	Lymphoepithelial cyst	Unilateral
7	Nikumbh et al.	2017	India	Multiple epidermal inclusion cysts	Unilateral
8	Hsu et al.	2017	Taiwan	Epidermoid cyst	Unilateral
9	Jain et al.	2017	India	Epidermoid cyst	Unilateral
10	Fernandes et al.	2018	India	Squamous inclusion cyst	Unilateral
11	Altindal and Unal	2019	Turkey	Epidermoid cyst	Unilateral
12	Bouatay et al.	2019	Tunisia	Epidermoid cyst	Unilateral
13	Kusuma et al.	2021	India	Epidermoid cyst	Unilateral
14	Moon et al.	2022	Korea	Lymphoepithelial cyst	Unilateral
15	Rahim et al.	2023	UK	Retention cyst	Unilateral
16	Shah et al.	2025	India	Lymphoepithelial cysts	Bilateral
17	Our case	2025	Japan	Retention cyst	Bilateral

*Note:* Previously reported cases of palatine tonsil cysts are summarized and compared with the present case.

Bilateral palatine tonsillar cysts diagnosed as retention‐type lesions have not been well documented in the existing literature. In this context, the present case represents one of the very few reported cases of bilateral palatine tonsillar cysts and provides novel clinical and pathological insights into this rare entity.

Endoscopic findings of palatine tonsillar cysts typically include smooth‐surfaced, well‐circumscribed lesions without ulceration, which may mimic benign or malignant tonsillar tumors [5, 7, 17]. Therefore, preoperative differentiation based solely on clinical findings can be challenging. Radiological evaluation is crucial for their assessment. Previous reports have described cystic tonsillar lesions appearing isointense or hypointense on T1‐weighted images and hyperintense on T2‐weighted images, without diffusion restriction on diffusion‐weighted imaging [8–10]. The imaging characteristics observed in the present case are consistent with these findings and support a benign cystic etiology.

A definitive diagnosis relies on histopathological examination. Reported cases are characterized by cyst walls lined with stratified squamous epithelium, often associated with underlying lymphoid tissue, indicating a tonsillar origin [2, 3, 12]. These findings emphasize the importance of pathological confirmation, even when clinical and radiological findings suggest a benign process.

Management strategies for palatine tonsillar cysts depend on symptom severity, lesion size, and diagnostic uncertainty. Asymptomatic lesions may be managed conservatively, whereas symptomatic cysts or those with uncertain malignant potential are generally treated surgically [1, 6, 11]. Endoscopic excision under general anesthesia has been reported as a safe and effective approach with favorable outcomes and low recurrence rates [3, 8, 9]. In the present case, complete endoscopic excision resulted in symptom resolution, and no recurrence was observed during the follow‐up.

This case highlights the importance of careful endoscopic evaluation, appropriate imaging, and histopathological confirmation in the diagnosis and management of rare bilateral palatine tonsillar cysts.

## Funding

This study received no external funding.

## Consent

Comprehensive treatment consent, including publication, was obtained.

## Conflicts of Interest

The authors declare no conflicts of interest.

## Data Availability

The data that support the findings of this study are not publicly available due to privacy or ethical restrictions.
